# Double encephalocele in a four-year-old girl: A case report with literature review

**DOI:** 10.12669/pjms.40.12(PINS).10964

**Published:** 2024-12

**Authors:** Ahtesham Khizar, Hassaan Zahid, Manal Khan, Abdul Rahim Farooq, Muhammad Aqeel Natt

**Affiliations:** 1 Ahtesham Khizar, MBBS, FCPS (Neurosurgery), Department of Neurosurgery Unit-I, Punjab Institute of Neurosciences, Lahore, Pakistan; 2 Hassaan Zahid, MBBS, FCPS (Neurosurgery), MS (Pediatric Neurosurgery), Department of Neurosurgery Unit-I, Punjab Institute of Neurosciences, Lahore, Pakistan; 3 Manal Khan, MBBS, Department of Neurosurgery Unit-I, Punjab Institute of Neurosciences, Lahore, Pakistan; 4 Abdul Rahim Farooq, MBBS Aga Khan University Medical College, Karachi, Pakistan; 5 Muhammad Aqeel Natt, MBBS, FCPS (Neurosurgery), Department of Neurosurgery Unit-I, Punjab Institute of Neurosciences, Lahore, Pakistan

**Keywords:** Encephalocele, Meningocele, Neural tube defects, Folic acid deficiency, Developing country

## Abstract

Encephalocele is a congenital neural tube defect (NTD). The pathophysiology of the NTDs is exceedingly complex. Numerous explanations have been proposed to explain it. Double encephaloceles are highly unusual. There have only been fifteen previously reported cases of double encephalocele in the medical literature, with this index case being the oldest and first from Pakistan. A four-year-old girl presented with two occipital scalp swellings from infancy. The occipital swelling measured about 7x5x3 cm, while the suboccipital swelling measured about 7x9x5 cm. The skin over both the swellings was intact. Following a thorough history, physical examination, and radiological investigations, surgical excision and repair was performed. Postoperative recovery was uneventful. She did not develop hydrocephalus until the three month follow-up.Double encephalocele is a rare entity. The multisite closure theory appears to be the most plausible explanation for the development of multiple NTDs. The management of double encephalocele requires a case based approach.

**Abbreviations:**
NTD: Neural tube defect,
CT: Computed tomography,
MRI: Magnetic resonance imaging,
CSF: Cerebrospinal fluid.

## INTRODUCTION

An encephalocele is a congenital neural tube defect (NTD) caused by failure of the cranial part of the developing neural tube to close, resulting in herniation of cranial contents via a defect in the skull. Encephaloceles are uncommon NTDs, affecting one in every 5,000 infants globally, with 70% being occipital.^1^ The pathophysiology of the NTDs is extremely complicated, including intricate interactions between genes, environment, and nutrition. Multiple hypotheses have been offered to explain neural tube formation using experimental models.^2^ Based on defect site, encephaloceles are classified as: i) occipital, ii) suboccipital, iii) sincipital (fronto-ethmoidal), iv) basal (trans-sphenoidal, trans-ethmoidal, spheno-ethmoidal, and spheno-orbital), and v) parietal.^3^ Double encephalocele is extremely rare; the majority of them involve the occipital or suboccipital region.^4^ There are only fifteen previously reported cases of double encephalocele in the medical literature, and this index case is the oldest and the first to be reported from Pakistan. We present the following case in accordance with the CARE-guidelines.^5^

## CASE PRESENTATION

**Fig.1:**
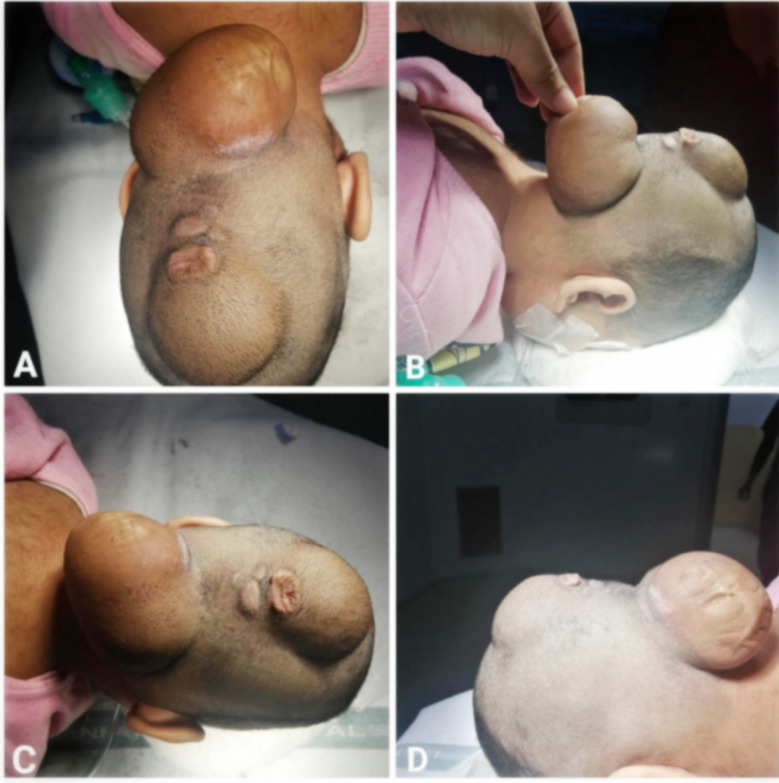
Double encephalocele, A&C: Superior views, B&D: Right lateral and left lateral views.

**Fig.2:**
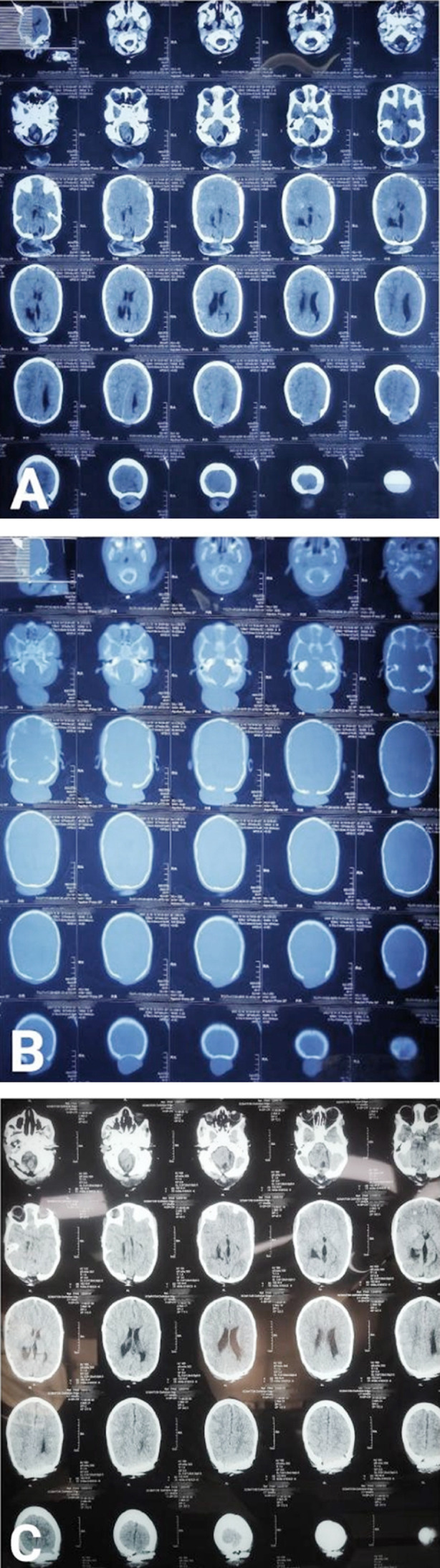
A: Preoperative CT brain plain axial view shows double encephalocele and bony defects, B: CT bone window shows bony defects, C: Postoperative CT brain plain axial view shows bony defect closure by cranioplasty and no hydrocephalus.

**Fig.3:**
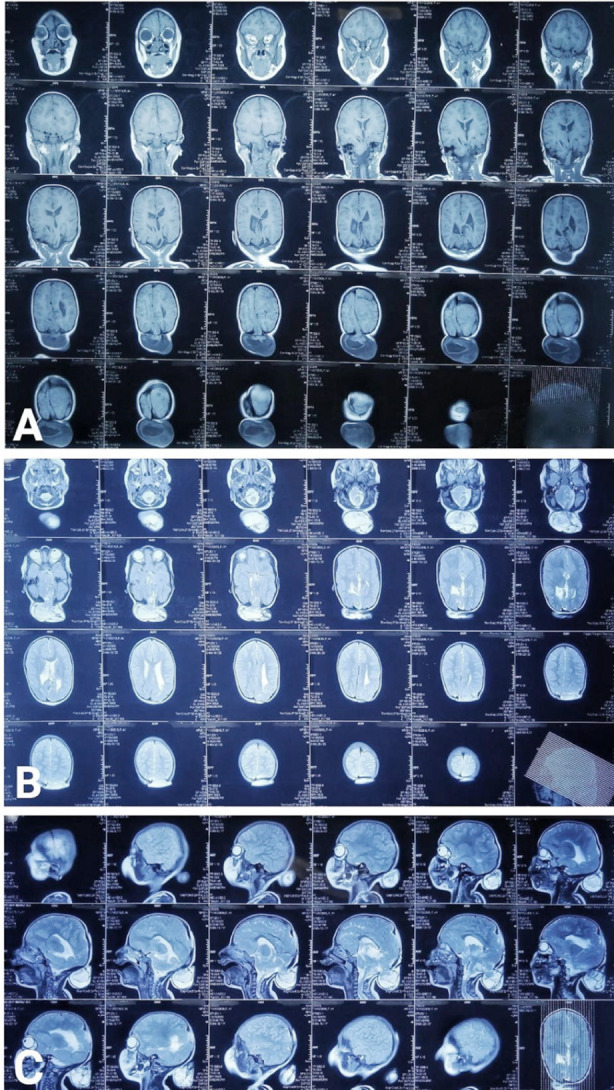
A: MRI brain axial T1WI showing occipital and suboccipital encephaloceles, B: Axial T2WI showing both the encephaloceles, C: Sagittal T2WI showing small occipital and large suboccipital encephalocele.

**Fig.4:**
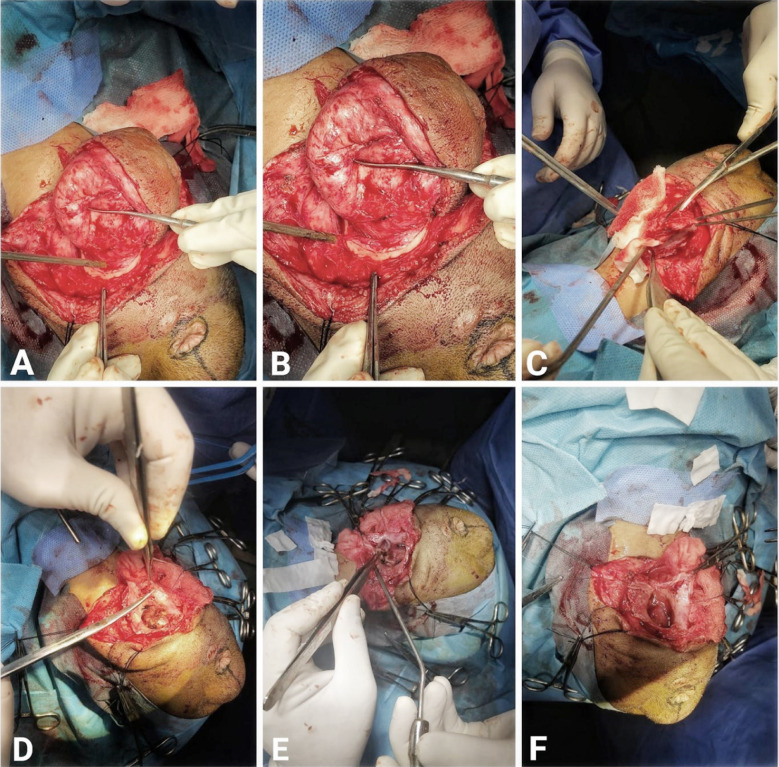
A&B: Exposure of the sac of suboccipital encephalocele, C,D&E: Sac opening F: After removal of devitalized neural tissue.

**Fig.5:**
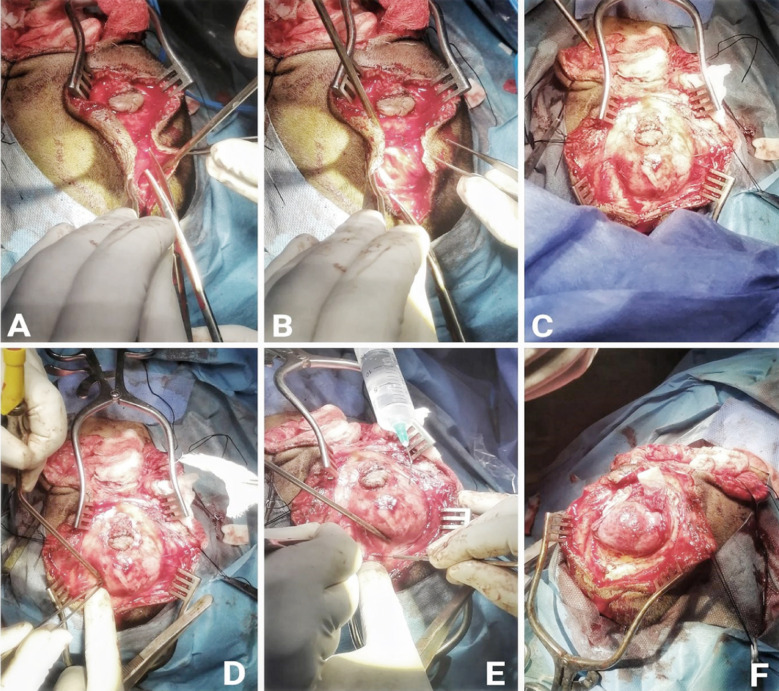
A&B: Dissection of occipital encephalocele, C,D&E: Sac exposure, F: Viable brain tissue inside sac.

A four years old girl came to us in December 2023 as an outpatient with a history of two occipital scalp swellings since birth. According to her mother, she was born at term in a small local hospital, and they did not seek additional medical care for her occipital swellings. On examination, the occipital swelling measured around 7x5x3 cm, whereas the suboccipital swelling measured about 7x9x5 cm. Overlying skin was intact over both the swellings, and a slight indentation was visible on the bottom edge of the occipital swelling. (Fig. 1,A,B,C&D) A bony defect was palpable around the occipital swelling. Transillumination was negative in both the swellings. Computed tomography (CT) brain plain with bone window and magnetic resonance imaging (MRI) brain plain with MR venogram were performed. CT showed both the encephaloceles and the bony defects. (Fig. 2,A&B) MRI brain showed soft tissue details (Fig. 3,A,B&C) whereas MR venogram showed details of the dural venous sinuses. The patient underwent surgical excision and repair for both the encephaloceles. During surgery, the suboccipital encephalocele was excised and repaired first. (Fig. 4,A,B,C,D,E&F) The sac contained cerebrospinal fluid (CSF) and devitalized neural tissue which was removed and dural repair was performed. Then occipital encephalocele was excised and repaired. (Fig. 5,A,B,C,D,E&F) Normal brain tissue was present inside the sac and the posterior part of the superior sagittal sinus was present on the medial edge, draining into the confluence of sinuses. Following dural repair, cranioplasty was also performed by using a titanium mesh plate. Postoperative recovery was uneventful. Postoperative CT brain plain showed no hydrocephalus. (Figure. 2,C) She was discharged home on the third day and at her follow-up visit after a week she had developed a cystic swelling on the suboccipital encephalocele site. We aspirated about 15 ml cystic fluid and applied a tight crepe bandage. There was no recurrent collection after that, and the bandage was removed on her second week of follow-up. She did not develop hydrocephalus until the three month follow-up.

## DISCUSSION

Encephaloceles are uncommon, with an incidence of 0.8 to 5 per 10,000 live births globally.^6^,^7^ Male and female carried the same incidence.^6^ Encephaloceles are typically solitary, with occipital encephaloceles being more common in general.^6^,^8^ Double encephaloceles are extremely uncommon, with only a few cases reported in the literature.^6^ Our literature review, conducted using the PubMed and Google Scholar databases, identified fifteen cases, as indicated in Table-I, with no cases reported on PakMediNet, making this the first case report of double encephalocele from Pakistan.

**Table-I:** Details of literature review on double encephalocele.

S. No.	Author	Year	Age/Gender	Diagnosis	Outcome
1	Goyal PK et al.^12^	2010	3 months/M	Double barrel meningomyelocele, 5x6 cm and 5.5x6.5 cm	Good recovery, no hydrocephalus till 2 year follow-up
2	Singh DK et al.^9^	2012	5 months/M	Double suboccipital meningoencephalocele, 7x6x6 cm and 4x3x3 cm	No neurological sequelae
3	Ramdurg SR et al.^13^	2014	6 months/M	Double encephalocele (one atretic and other occipital)	Immediate good outcome
4	Canaz H et al.^8^	2015	1 day/M	Supra- and infra-torcular double occipital encephalocele, 9x8 cm and 10x9 cm	No hydrocephalus, developmental delay till 3 year follow-up
5	Sharma S et al.^7^	2016	2 months/M	Double encephalocele, 2.2x2.8 cm and 2.5x2.1 cm	VP shunt for hydrocephalus, no other issues till 3 months follow-up
6	Menekse G et al.^14^	2017	2 weeks/F	Double encephalocele	Encephalomalacia, no hydrocephalus till 3 months follow-up
7	Yhoshu E et al.^15^	2018	2 years/M	Double cranial encephalocele, 4x4 cm and 2x2 cm, porencephalic cyst and hydrocephalus	VP shunt for hydrocephalus, slight improvement in milestones at 1 year follow-up
8	Garg D et al.^16^	2019	Newborn/M	Double encephalocele, 4x4 cm and 2x2 cm	No neurological sequelae
9	da Silva AJF et al.^17^	2020	Newborn/F	Amniotic band syndrome with double frontal encephaloceles, 6x4 cm and 6x5.5 cm	VP shunt for hydrocephalus
10	Shah CK et al.^6^	2021	In-utero/M	Occipital and parietal encephalocele	VP shunt for hydrocephalus, delayed milestones at 2 years of age
11	Abdulwahid AT et al.^4^	2023	2 months/F	Microcephaly with double occipital encephalocele, 5x5 cm and 4.5x5 cm	No neurological sequelae
12	Singh AP et al.^18^	2023	Newborn/F	Double encephalocele	VP shunt for hydrocephalus
13	Elmaghrabi M et al.^19^	2023	-	Reported 3 cases of double encephalocele	1 case developed hydrocephalus
14	Index case	2024	4 years/F	Double encephalocele, 5x4 cm and 7x6 cm	No hydrocephalus till 3 months follow-up

Neural tube genesis and closure need complicated cellular, extracellular, and intracellular processes. There are two basic ideas on neural tube closure. The commonly recognized hypothesis is that neural tube closure occurs in a continuous, bidirectional process that begins in the mid-cervical region and advances in a zipper-like pattern both rostrally and caudally, with the cranial and caudal neuropores closing last. There are several flaws in this relatively rudimentary ’zipper concept’. This idea suggests that meningomyeloceles are more commonly seen at the most cranial or caudal ends, however it does not account for cervical meningomyelocele, multiple NTDs, or double encephalocele.^9^

The findings of multiple meningoceles can be explained at different levels by the multisite closure theory put out by Van Allen et al.^10^ and Nakatsu et al.^11^ According to the multisite neural tube closure model, human normal neural tubes have several closure points, or “zippers”. Presumably, one or more genes regulate these zippers; mutations in these genes would result in neural tube abnormalities in the vicinity of the affected zipper.^9^ This could explain why double NTDs develop in embryogenesis at different locations similar to our index case.

The mainstay of treatment for encephalocele is surgical. This surgery consists of incising the sac, amputating the extra tissue to the level of the surrounding skull, dural closure, and skin closure. In general, infants born with an occipital encephalocele surrounding the brain have a poor prognosis. In addition to the contents of the sac, the extent of the lesion influences the long-term prognosis.^6^ In our case, parents of the girl were satisfied with the surgical treatment of their daughter.

## CONCLUSION

Double encephalocele is a rare condition. The multisite closure theory appears to be the most plausible explanation for the development of multiple NTDs. The management of double encephalocele is a challenge that requires unique solutions for each instance.

### Consent for publication:

Consent was obtained from the father of the girl for publication of this case report and the accompanying images.

### Authors’ Contribution:

**AK:** Conception and Design of study, Data acquisition, Manuscript writing and Literature review.

**HZ:** Critical review, Literature search and review, Is responsible and accountable for the accuracy and integrity of the work.

**MK and ARF:** Literature review and Manuscript writing.

**MAN:** Supervision and Critical review.

All the authors have read and approved the final manuscript.

## References

[1] Zahid S, Khizar A (2021). Giant occipital encephalocele:a case report, surgical and anesthetic challenge and review of literature. Egypt J Neurosurg.

[2] Yadav JK, Khizar A, Yadav PK, Mustafa G, Bhatti SN (2019). A case report of triple neural tube defect:revisiting the multisite closure theory. BMC Surg.

[3] Suwanwela C, Suwanwela N (1972). A morphological classification of sincipital encephalomeningoceles. J Neurosurg.

[4] Abdulwahid AT, Al-Obaidi AD, Al-Obaidi MN, Hashim HT (2023). Double encephalocele with an excellent outcome postoperatively:A case report from Iraq. eNeurologicalSci.

[5] Riley DS, Barber MS, Kienle GS, Aronson JK, von Schoen-Angerer T, Tugwell P (2017). CARE guidelines for case reports:explanation and elaboration document. J Clin Epidemiol.

[6] Shah CK, Lee RY, Jeph S (2021). In-utero Diagnosis of Double Encephalocele - Imaging Features and Review of Literature. J Radiol Case Rep.

[7] Sharma S, Ojha BK, Chandra A, Singh SK, Srivastava C (2016). Parietal and occipital encephalocele in same child:A rarest variety of double encephalocele. Eur J Paediatr Neurol.

[8] Canaz H, Ayçiçek E, Akçetin MA, Akdemir O, Alataş I, Özdemir B (2015). Supra- and infra-torcular double occipital encephalocele. Neurocirugia (Astur).

[9] Singh DK, Singh N, Kumar P (2012). Double suboccipital meningoencephalocele:a unique case report. Pediatr Neurosurg.

[10] Van Allen MI, Kalousek DK, Chernoff GF (1993). Evidence for multi-site closure of the neural tube in humans. Am J Med Genet.

[11] Nakatsu T, Uwabe C, Shiota K (2000). Neural tube closure in humans initiates at multiple sites:evidence from human embryos and implications for the pathogenesis of neural tube defects. Anat Embryol (Berl).

[12] Goyal PK, Singh D, Singh H, Tandon M (2010). Suboccipital double barrel twin meningocoele:Another new theory?. J Pediatr Neurosci.

[13] Ramdurg SR, Gubbi S, Odugoudar A, Kadeli V (2014). A rare case of split pons with double encephalocoele, dermal sinus tract, and lipomeningomyelocele:a case report and review of literature. Childs Nerv Syst.

[14] Menekse G, Celik H, Bayar MA (2017). Giant Parietal Encephalocele with Massive Brain Herniation and Suboccipital Encephalocele in a Neonate:An Unusual Form of Double Encephalocele. World Neurosurg.

[15] Yhoshu E, Dash V, Bawa M (2018). Double Encephalocele:An Unusual Presentation. J Pediatr Neurosci.

[16] Garg D, Singh AP, Tanger R, Gupta AK (2019). Double encephalocele arising from single bone defect:A rare case. Journal of Clinical Neonatology.

[17] da Silva AJF, Silva CSME, Mariano SCR (2020). Amniotic band syndrome with double encephalocele:A case report. Surg Neurol Int.

[18] Singh AP, Kumar A, Barolia DK, Solanki N, Bathia HV (2023). Double encephalocele:A rare neural tube defect. J Pediat Neurosci.

[19] Elmaghrabi M, Arab A, El Awady M, Mourad M (2023). Management of encephalocele in infants:a 5-years retrospective study in Benha, Egypt. Benha Medical Journal.

